# Choropleth map legend design for visualizing community health disparities

**DOI:** 10.1186/1476-072X-8-52

**Published:** 2009-09-24

**Authors:** Robert G Cromley, Ellen K Cromley

**Affiliations:** 1 Department of Geography, University of Connecticut, Storrs, Connecticut, USA; 2 The Institute for Community Research, Hartford, Connecticut, USA

## Abstract

**Background:**

Disparities in health outcomes across communities are a central concern in public health and epidemiology. Health disparities research often links differences in health outcomes to other social factors like income. Choropleth maps of health outcome rates show the geographical distribution of health outcomes. This paper illustrates the use of cumulative frequency map legends for visualizing how the health events are distributed in relation to social characteristics of community populations. The approach uses two graphs in the cumulative frequency legend to highlight the difference between the raw count of the health events and the raw count of the social characteristic like low income in the geographical areas of the map. The approach is applied to mapping publicly available data on low birth weight by town in Connecticut and Lyme disease incidence by town in Connecticut in relation to income. The steps involved in creating these legends are described in detail so that health analysts can adopt this approach.

**Results:**

The different health problems, low birth weight and Lyme disease, have different cumulative frequency signatures. Graphing poverty population on the cumulative frequency legends revealed that the poverty population is distributed differently with respect to the two different health problems mapped here.

**Conclusion:**

Cumulative frequency legends can be useful supplements for choropleth maps. These legends can be constructed using readily available software. They contain all of the information found in standard choropleth map legends, and they can be used with any choropleth map classification scheme. Cumulative frequency legends effectively communicate the proportion of areas, the proportion of health events, and/or the proportion of the denominator population in which the health events occurred that falls within each class interval. They illuminate the context of disease through graphing associations with other variables.

## Background

Disparities in health outcomes across communities are a central concern in public health and epidemiology. Methods of exploratory data analysis and geovisualization are commonly used to provide insight into the patterns of the health outcomes. Choropleth maps of health outcome rates calculated for administrative units at a variety of scales have long been used in public health and epidemiology, and their preparation is now supported by geographic information systems. These maps display the spatial properties of a data distribution but do not directly present the associated statistical distribution of the data. In addition, choropleth maps usually show the geographical distribution of health outcomes in individual layers that not link to spatial patterns of other social factors like income which may play a role in health disparities.

The purpose of this research is to improve the communicative efficiency of choropleth maps of health outcomes through an enhanced legend design. This design approach uses different combinations of cumulative frequency graphs in the map legend to highlight alternatively the underlying statistical distribution, the difference between the raw count of the numerator and the raw count of the denominator, and contextual information regarding characteristics of the areas being mapped. The approach is applied to mapping publicly available data on low birth weight and Lyme disease incidence by town in Connecticut.

Despite their wide use, the limitations of choropleth maps are well-known [1]. First, there is the numbers issue. Mapping absolute counts can be misleading because spatial units vary in area and number of observations. One solution is to standardize by calculating a density, rate, ratio, or proportion. This approach is commonly used with health event data. Health rates are also sometimes adjusted by age-sex standardization and/or smoothing for comparison mapping purposes. Direct age-sex standardization is only possible, however, if the health events in the numerator are reported for multiple age-sex strata. Indirect standardization methods based on a standard set of age-specific rates to the population of interest may be used but have their own limitations [2]. In many cases, health data are not reported for these finer strata, the denominator data for the age-sex strata are not available, or an age-specific rate is reported. Even when standardization is possible, it can also mask important differences in the distribution of the numerator and the denominator. Standardization presents the risk of an outcome but obscures the absolute number of outcomes present.

Second is the area issue. The spatial units have boundaries that arbitrarily partition the underlying distribution of interest. Portraying standardized rates can be misleading if the denominator is not the area of the spatial unit. Larger areas dominate the visual display, and a sparsely populated, large area unit will distort the communication of the overall health risk. One solution is to use a cartogram to transform the size of the spatial unit so that it is proportional to the denominator and not to the geographic area [1]. This often results in non-contiguous cartograms that destroy the topology and mask where events are occurring or contiguous cartograms such as the Gastner-Newman cartogram [3] that preserves contiguity but distorts the shape of units. A recent cognitive study [4] has found that cartograms could be an effective means of portraying public health data if the map reader has or is given a sufficient background on the nature of cartograms.

Third are the classification issues. Most accuracy issues in choropleth mapping are related to the data classification step in classed maps [5]. Because the categorization processes of the human brain limit the number of graphic values that can be discerned simultaneously, traditional choropleth maps break the data distribution into a small number of class intervals limiting the number of graphic values in the map. Because numerous partitions of the data distribution exist even for maps using a few geographic units and a small number of classes, it is relatively easy to manipulate the class breaks of any statistical distribution and its corresponding spatial representation [6]. Unclassed choropleth maps, in which individual numeric values are transformed into graphic values [7], have also been developed to eliminate the loss of information due to classification and the chance to manipulate the display through choice of classification. However it is difficult to extract the underlying statistical distribution even in unclassed choropleth maps. Several authors [8,9] have noted that lightness (or value) is a good visual variable for perceiving the ordinal scale relationships that are necessary for understanding spatial pattern, but it is only a moderately effective variable for perceiving numerical data.

**Figure 1:**
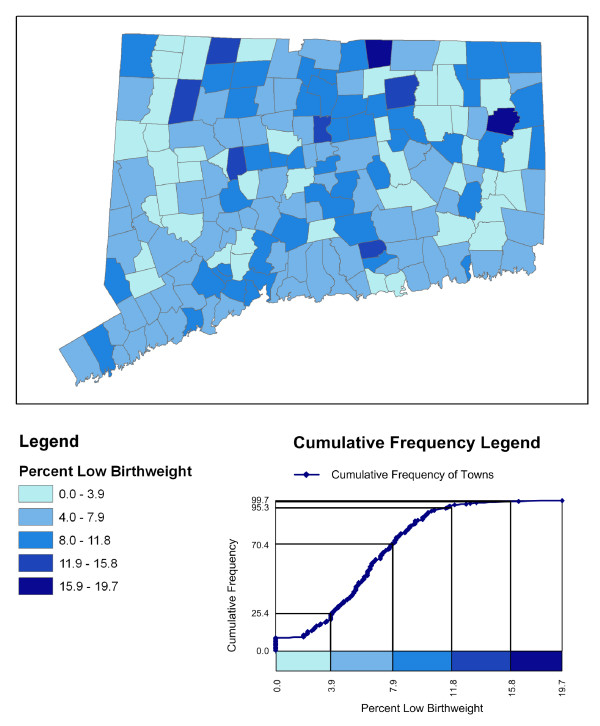
**A choropleth map depicting the percent of low birthweight with alternative legend designs**. An equal interval classification is used to portray the spatial distribution of the percent of low birthweight births by town in Connecticut. The bottom-left diagram is a standard legend connecting the colors in the map to the range of data they represent. The bottom-right diagram is a cumulative frequency legend that also depicts the cumulative percentage of towns associated with each data interval on the map.

It is the map legend that links the graphic value in the body of the map with its corresponding numeric value. GIS software packages make it easy for users to classify choropleth maps and generate standard legends. However, these legends are limited in the information they provide regarding the statistical distribution. For classed maps, a series of legend boxes, ordered either vertically or horizontally, associate each class symbolization box with its range of numeric values. Figure 1 presents an equal interval classed choropleth map depicting the percent of low birthweight births with a standard legend shown at the lower-left of the figure. The map reader can tell that the lightest blue tone representing the lowest class interval has a data range between 0.0 percent and 3.9 percent low birthweight births. Although one can readily examine the range for each interval, it is difficult to extract information regarding the nature of the statistical distribution.

Kumar [10] suggests supplementing standard map legends with a graph of the frequency distribution of the rates or a histogram. A frequency distribution shows the statistical distribution of rates to complement the spatial distribution revealed in the map. Frequency distribution graphs have been incorporated in a variety of health atlases [11,12]. However, frequency histograms are sensitive to number of classes and class breaks and therefore change in shape as the map classifications change even though the statistical distribution has not changed.

Cumulative frequency distributions are an alternative for supplementing standard map legends. A cumulative frequency curve of the distribution, unlike the frequency histogram, is the same regardless of the number of class intervals or the map classification scheme [13,14]. Furthermore, Andrienko and Andrienko [15] have demonstrated that more than one cumulative frequency curve can be displayed in a graph referenced to a common variable. For these reasons, cumulative frequency legends are a useful choice for restructuring and enhancing the choropleth map legend [14]. Alexandersson [16] first used a cumulative frequency diagram as part of the legend design associated with a map, in his map displaying the industrial structure of the United States. The versatility of the cumulative frequency approach ensures that distributions of both numerators and denominators of rates can be included as well as the distributions of related socioeconomic variables of interest.

## Methods

The data for the research include a shapefile of town boundaries for the 169 towns of Connecticut. Towns are considered county subdivisions by the Census. Towns are the basic units of local government in the state. Every location in Connecticut is in one and only one town. There are no unincorporated areas in the state. Because the towns have relatively small areas, they are commonly used to map health data in Connecticut, a state with only 8 counties.

Two sets of health data are mapped: data on low birthweight births by town in 1998 and data on Lyme disease cases by town for 2001. These data are publicly available and were obtained from the Connecticut Department of Health website. These data were chosen because they provide information on very different types of health problem affecting the state's population. Low birth weight is defined as less than 2500 g (5lb 8oz). The causes of low birth weight are complex and include behavioral, sociodemographic, and medical care access factors. The rate for low birth weight is given in percent. Lyme disease represents a very different kind of health problem. Lyme disease is a multistage multisystem vector-borne disease caused by the bacterium *Borrelia burgdorferi*. In Connecticut, *Ixodes scapularis *(the black-legged or deer tick) is the vector responsible for transmitting the disease to humans. The rate for Lyme disease is given per 100,000 population. Exposure to Lyme disease in Connecticut is primarily peridomestic so mapping cases based on town of residence is appropriate for this study.

Finally, one set of socioeconomic data on the number of people living below the poverty level by town in 2000 was used. These data were downloaded from the U.S. Census Bureau website. Data on poverty were used because income has been highlighted as a factor affecting health disparities. Nevertheless, data on any variable available at the town level and believed to be relevant to a particular disease process could be used.

Constructing a cumulative frequency legend begins with a diagram in which the range of the variable being mapped is given along the horizontal axis of the diagram and the cumulative frequency variable in percents is given along the vertical axis. More than one cumulative frequency variable can be graphed on the vertical axis. For classed choropleth maps, a horizontal bar is inserted under the cumulative frequency diagram. In essence, this bar turns the traditional choropleth map legend on its side. The bar is segmented corresponding to the length of each classification interval and each segment is colored in the representation of that class in the map. The class limits are used as the anchor points for each segment and a set of vertical and horizontal lines (here called "projection lines") can be superimposed over the diagram to associate cumulative frequency values on the vertical axis with their respective class limits on the horizontal axis. An example cumulative frequency legend for the equal interval map in Figure 1 is shown in the lower-right portion of that figure.

A number of software packages are available that support choropleth mapping and creating cumulative frequency legends for maps. In this research a GIS application using ArcGIS 9.2 (ESRI) mapped the rate data for low birthweight births and for Lyme disease using different choropleth map classification schemes. Next standard software functions were used to prepare cumulative frequency map legends. The attribute tables of low birthweight data and Lyme disease data were exported from ArcGIS and imported into an Excel (Microsoft) spreadsheet. In Excel, the towns were ordered from lowest to highest based on their low birthweight rates and their Lyme disease rates. Then, variables were created showing the cumulative frequencies of the number of towns, the numerators and denominators of the rates, and the population below the poverty level and the total population in each town. The cumulative frequencies were based on the ordered rates of the health events being mapped, one set of cumulative frequencies for the ordered low birthweight rates and one set of cumulative frequencies for the ordered Lyme disease rates. Then, the chart wizard in Excel was used to graph the cumulative frequency of interest against the ordered health rate of interest. Finally, the graph was exported into Visio (Microsoft), a graphics program, and the horizontal bar with the choropleth map class interval breaks and the projection lines were added.

## Results and Discussion

### Observational Units and Ogives

As discussed above, the choropleth map in Figure 1 shows the contrast between a standard legend and a cumulative frequency legend using the number of observational units in the map as the cumulative frequency variable (the "ogive"). The data range for each class interval in the cumulative frequency legend is given using both numbers and length--a high-level perceptual task for visually decoding information [17]-whereas the range for each class interval in the standard legend is only given by numbers. Because this ogive is based on the cumulative frequency of observational units, the map reader can easily discern that 25.4% of the geographic units are in the lowest class interval (and that a number of these units have 0.0% rate) by examining the projection lines associated the upper limit of that class interval. The standard legend gives no indication of the percent of observations in any class. The reader would need to count the number of units on the map shaded the same interval color to evaluate the percentage of town falling within that interval.

### Numerators and Denominators

Counts are not used in mapping to make an inference about the risk of health outcomes because it is expected that units with larger populations would have a higher number of events. However, maps of rates may mask the spatial pattern in risk if the rates are based on populations of very different sizes. The small numbers problem arises whenever a small population at risk is involved as the rate denominator. A tension also exists between the relative and absolute view of health outcomes in which the risk of an outcome must be weighted against the frequency of occurrence of the numerator of the rate. High risk areas are a warning to residents and a chance to understand the context of the disease, but the location of clinics to treat health outcomes may depend more on the location of outcome occurrences than it does on the level of risk. Besides understanding the distribution of rates it is also necessary to investigate the distribution of the numerators and denominators associated with those rates.

A numerator/denominator cumulative frequency legend is designed in a manner similar to the basic ogive cumulative frequency legend. The difference now is that rather than a single graph of the frequency of occurrence of spatial units in a class interval, two graphs are given showing the cumulative frequency of the numerator values (the health outcome) and the cumulative frequency of the denominator values (population at risk) respectively. The focus of these graphs is now on the distribution of health events (the numerator) versus the distribution of the population within which the event occurs (the denominator) rather than on the geographic units themselves as in the ogive version. In this legend, the denominator curve will always be above the numerator curve because the order for the graphs is determined by the rate values which are ordered from low to high along the horizontal axis.

**Figure 2:**
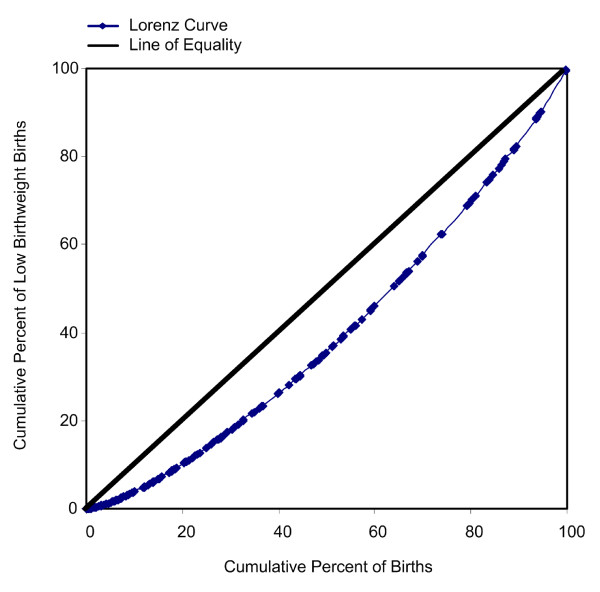
**A Lorenz Curve diagram depicting the distribution of low birthweight births against that of births**. Towns are first rank ordered by their percent low birthweight. For each town, the cumulative percent of low birthweight births and the cumulative percent of births are plotted as coordinates to form the Lorenz Curve. The deviation of the Lorenz Curve from the line of equality denotes a concentration of low birthweight births within the larger population of births.

Numerator/denominator cumulative frequency legends are also an alternative to the Lorenz Curve for visualizing concentration. In a Lorenz Curve, the cumulative frequency of the numerator, given on the vertical axis, is plotted against the cumulative frequency of the denominator, given on the horizontal axis, as a coordinate of the curve [18]. By definition, a Lorenz Curve lies below the 45° line, the line of equality, representing equal proportions for all spatial units (if the vertical and horizontal axes are reversed, the Lorenz Curve always lies above this line). Figure 2 displays the Lorenz Curve that plots the cumulative frequency of low birthweight births against the cumulative frequency of births. The Lorenz Curve has become a standard method for analyzing health inequalities and disease patterns across geographic units [19-21]. As discussed above, the numerator/denominator legend plots two curves--the cumulative frequency of the numerator against the rate value for each spatial unit and the cumulative frequency of the denominator against the rate value for each spatial unit. The maximum difference between these curves is equal to the Index of Dissimilarity, described in standard statistical texts [18]. The Index of Dissimilarity measures the evenness with which a variable like number of deaths is distributed across a set of classes like age cohorts. The Index indicates whether or not deaths are concentrated in particular age cohorts. The Index of Dissimilarity ranges in value from 0.0 (perfectly uniform) to 100.0% (perfectly concentrated within a class).

In many social science applications, the classes are geographical units but the spatial arrangement of the units is not taken into account in the calculation of the index. Two study regions with the same Index of Dissimilarity could show different patterns of spatial autocorrelation in the distribution of areas that have high concentrations of the variable of interest. In this case, the cumulative frequency graph is enhanced by the accompanying map. The Index of Dissimilarity can be added to the cumulative frequency legend by adding a vertical line at the grand rate. The maximum difference is reached along the horizontal axis of the legend at the grand rate for the entire study region because the proportion of the total associated with the denominator is always greater than the proportion of the total associated with the numerator for individual rate values below the grand rate and vice versa for individual rates above the grand rate.

**Figure 3:**
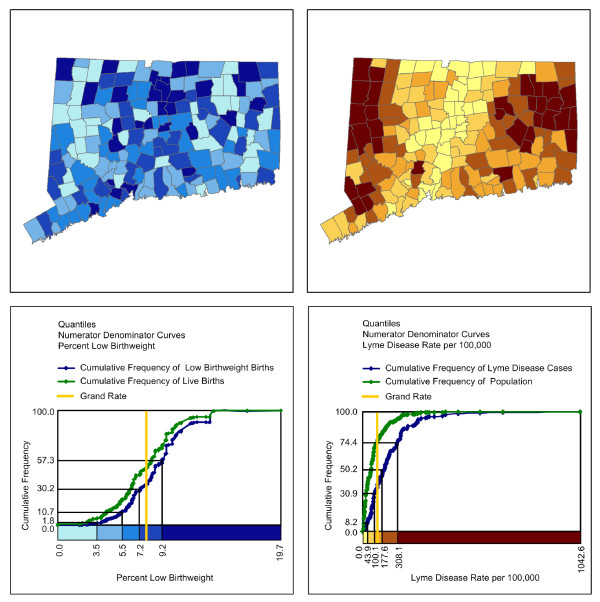
**Choropleth maps with numerator/denominator cumulative frequency legends**. A quantile classification in which an equal number of towns are in each data class is used to portray the spatial distribution of the percent of low birthweight births by town in Connecticut in the upper-left diagram and to portray the spatial distribution of Lyme disease per 100,000 population by town in Connecticut in the upper-right diagram. A numerator/denominator cumulative frequency legend associated with each map is presented beneath the respective map. In these legends, the cumulative frequency curve for numerator is given in blue and that for the denominator is given in green. The grand rate for each health outcome is given by a vertical yellow line that defines the maximum separation between the two curves for any value.

Maps of low birthweight rates and Lyme disease rates, each using a five-class quantile classification in which an approximately equal number of towns are in each class interval, can be compared along with their cumulative frequency numerator/denominator legends (Figure 3). The cumulative frequency legend for the map of low birth weight on the left reveals an approximately normal (s-shaped) distribution of low birthweight rates and low birth events. The numerator and denominator curves also follow each other relatively closely. The grand rate is 7.7% and the Index of Dissimilarity is only 14.45% suggesting that low birthweight births are relatively evenly spread across births classified by town in the state. The data show that low birthweight births occur in towns with large and small populations across the state. For low birth weight, 57.3% of low birthweight births in the numerator occurred in towns with low birthweight rates of 9.2% or less.

The cumulative frequency legend for the map of Lyme disease reveals a very different and highly positively skewed distribution. Most cases are in towns that are not large population centers. The grand rate is 102.0 cases per 100,000. The maximum gap on the vertical axis between numerator and denominator curves at the grand rate illustrates that there is more of a concentration of cases across populations classified by town given that the Index of Dissimilarity equals 38.50%. For Lyme disease, 74.4% of Lyme disease cases occurred in towns with relatively low rates.

**Figure 4:**
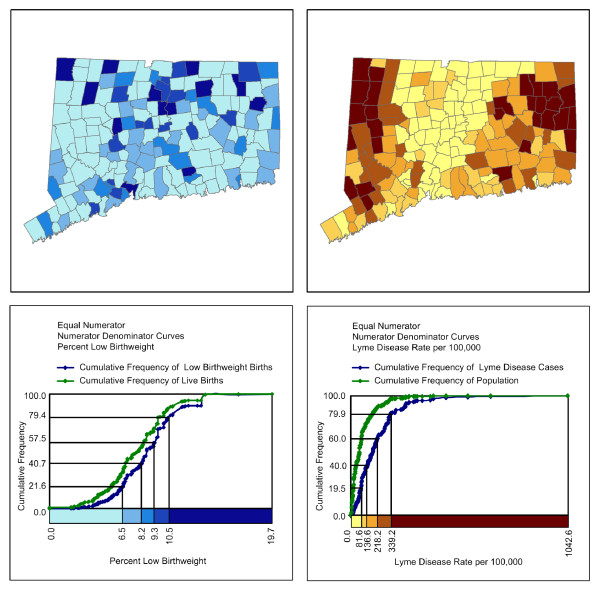
**Choropleth maps of health outcomes using an equal numerator classification**. In an equal numerator classification, approximately the same number of health outcome events are associated with each color representing a class interval in the map. This classification portrays the spatial distribution of the percent of low birthweight births by town in Connecticut in the upper-left diagram and portrays the spatial distribution of Lyme disease per 100,000 population by town in Connecticut in the upper-right diagram. A numerator/denominator cumulative frequency legend associated with each map is presented beneath the respective map.

The numerator/denominator cumulative frequency legend also suggests an alternative approach to standard data classification schemes in choropleth mapping. Instead of basing the map classification on the rate, it might be more effective to base the map classification on the distribution of the numerator. An equal numerator classification was developed that set the class intervals for the rate such that each interval contains an approximately equal number of health outcomes (Figure 4). The quantile maps based on equal numerators required a larger number of spatial units in the lowest class because not many health outcomes are associated with towns that have low rates. For low birth weight, 56.2% of the towns are in the lowest class based on an equal numerator classification. These 95 towns (shaded the lightest tone on the map) together accounted for 731 of all low birthweight births in the state while the 13 towns in the highest class (7.7% of all towns) accounted for 698 of all low birthweight births in the state, an almost equal number of low birthweight births. For Lyme disease, 34.3% of the towns are in the lowest class. These 58 towns together had 678 Lyme disease cases while the 28 towns in the highest class (16.6% of all towns) accounted for 698 Lyme disease cases. Overall, the equal numerator classification for Lyme disease is closer to having an equal number of towns in each class than the equal numerator classification for low birth weight because the cumulative frequency curve for low birthweight births was more similar in shape to its corresponding ogive than the cumulative frequency curve for Lyme disease cases was to its corresponding ogive.

### Community Context: Poverty Level

Cumulative frequency legends can also be used to display related socioeconomic variables rather than numerators and denominators of disease rates. The visual display of multivariate relationships between the mapped health rate and other associated variables has been examined using conditioned choropleth maps [22]. The focus here is on using the legend to display health disparities across social groups. Poverty level is used because income is frequently cited as one factor affecting health outcomes [23-26]. As an example, the population below the poverty level and the total population of each town were plotted against the ordered rates of low birth weight and Lyme disease. When curves other than the numerator or denominator of the mapped rate are used, one curve may or may not be above another curve. In the case of population below poverty versus total population, the relative positions of these two curves will depend on whether the population below or above the poverty level is more affected by the health events.

**Figure 5:**
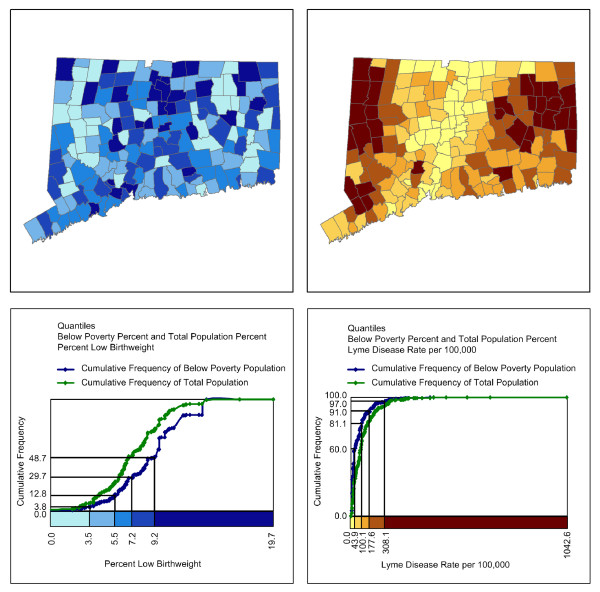
**Choropleth maps with contextual cumulative frequency legends**. A quantile classification is again used to portray the spatial distribution of the percent of low birthweight births by town in Connecticut in the upper-left diagram and to portray the spatial distribution of Lyme disease per 100,000 population by town in Connecticut in the upper-right diagram. A contextual cumulative frequency legend associated with each map is presented beneath the respective map. In these legends, the cumulative frequency curve for the population below the poverty level is given in blue and that for total population is given in green.

In Figure 5, a five-class quantile classification of rates is again used to map low birthweight births on the left and Lyme disease on the right. In this figure, however, the cumulative frequency legends now show the percent of poverty population and the percent of total population associated with each class interval on the map. In the legend showing the distribution of poverty across towns with different low birthweight rates, 51.3% of the poverty population is in towns with the highest low birthweight rates. The curve for the population below poverty is below the curve for total population, suggesting that the poverty population is concentrated in towns with high low birthweight rates.

The cumulative frequency legend showing poverty against Lyme disease rates, however, shows a very different picture. Here, 60.0% of the population below poverty is in towns in the lowest quintile of Lyme disease rates. Only 3.0% of the poverty population lives in towns in the highest quintile of Lyme disease rates. The quintile classification scheme has roughly the same number of towns in each class but the distribution of population below the poverty level in those towns is highly skewed. Although low income has been highlighted as a factor linked to health disparities, in this case we see that low income is not associated with all health problems.

## Conclusion

A previous cognitive study [14] has shown that an ogive-based legend can improve the map reader's ability to recognize of the nature of the statistical distribution associated with a choropleth map. It is hoped that cumulative frequency extensions of this legend design will be a useful supplement for health analysts who regularly use choropleth maps. They contain all of the information found in standard choropleth map legends and can be used with any choropleth map classification scheme. These legends also can be constructed using readily available software.

Cumulative frequency legends illuminate the context of disease through graphing associations with other variables. Although, income was featured in the cumulative frequency legends in this research, multiple contextual variables such as ethnicity and education could be overlaid in the same legend. It would also be possible to cumulate variables that are not tied to population. For example, the number of residential lots greater than a certain size in each town could used on the map of Lyme disease to investigate the degree to which land use patterns are associated with Lyme disease rates. Andrienko and Andrienko [15] note that one advantage of generalized cumulative frequency diagram is the ability to compare two or more variables in a single diagram without the prior selection of an object subset. They also note its advantage over other standard diagrams such as scatterplots. A cumulative frequency graph is more efficient than a thematic map such as the choropleth map with respect to the volume of data that can appear in one display. A separate map would be needed for each variable and the series of choropleth maps would need to be interpreted for visual comparisons to determine the underlying community context.

Despite reservations that researchers have regarding the choropleth map, it still remains the most frequently used map in public health [4]. The use of cumulative frequency legends can enhance the usefulness of choropleth maps for public health analysts. These legends make it possible to assess the number of geographical areas that fall within any class interval and the associated maps show where these areas are located. The legends might also help health analysts by showing that different health problems have different cumulative frequency signatures. It might be possible to find associations between health problems based on their cumulative frequency patterns. Finally, the choropleth map used to display the spatial pattern of risk coupled with the cumulative frequency legend used to provide contextual information on the communities of interest communicates valuable information on factors that may be associated with health disparities across areas within the larger study community.

## Competing interests

The authors declare that they have no competing interests.

## Authors' contributions

Both authors conceptualized the study, collected and mapped the data, constructed the legends and produced original and final drafts.
